# Sugar Responses of Human Enterochromaffin Cells Depend on Gut Region, Sex, and Body Mass

**DOI:** 10.3390/nu11020234

**Published:** 2019-01-22

**Authors:** Amanda L. Lumsden, Alyce M. Martin, Emily W. Sun, Gudrun Schober, Nicole J. Isaacs, Nektaria Pezos, David A. Wattchow, Dayan de Fontgalland, Philippa Rabbitt, Paul Hollington, Luigi Sposato, Steven L. Due, Christopher K. Rayner, Nam Q. Nguyen, Alice P. Liou, V. Margaret Jackson, Richard L. Young, Damien J. Keating

**Affiliations:** 1 College of Medicine and Public Health, Flinders University, Bedford Park SA 5042, Australia; amanda.lumsden@flinders.edu.au (A.L.L.); alyce.martin@flinders.edu.au (A.M.M.); emily.sun@flinders.edu.au (E.W.S.); 2 Nutrition & Metabolism, South Australian Health and Medical Research Institute (SAHMRI), Adelaide, SA 5000, Australia; gudrun.schober@sahmri.com (G.S.); nicole.isaacs@adelaide.edu.au (N.J.I.); nektaria.pezos@adelaide.edu.au (N.P.); richard.young@adelaide.edu.au (R.L.Y.); 3 Adelaide Medical School, The University of Adelaide, Adelaide SA 5005, Australia; chris.rayner@adelaide.edu.au (C.K.R.); QuocNam.nguyen@health.sa.gov.au (N.Q.N.); 4 Department of Surgery, Flinders Medical Centre, Bedford Park, SA 5042, Australia; david.wattchow@flinders.edu.au (D.A.W.); dayan.defontgalland@flinders.edu.au (D.d.F.); philippa.rabbitt@gmail.com (P.R.); paul.hollington@sa.gov.au (P.H.); luisposato@gmail.com (L.S.); steven.due@gmail.com (S.L.D.); 5 NHMRC Centre of Research Excellence in Translating Nutritional Science to Good Health, The University of Adelaide, Adelaide, SA 5005, Australia; 6 Cardiovascular, Metabolic, and Endocrine Diseases Research Unit, Pfizer Worldwide Research and Development, Cambridge, MA 02139, USA; aplioudvm@gmail.com (A.P.L.); vmjackson001@gmail.com (V.M.J.)

**Keywords:** serotonin, 5-hydroxytryptamine, 5-HT, glucose, enterochromaffin, obesity, duodenum, colon

## Abstract

Gut-derived serotonin (5-HT) is released from enterochromaffin (EC) cells in response to nutrient cues, and acts to slow gastric emptying and modulate gastric motility. Rodent studies also evidence a role for gut-derived 5-HT in the control of hepatic glucose production, lipolysis and thermogenesis, and in mediating diet-induced obesity. EC cell number and 5-HT content is increased in the small intestine of obese rodents and human, however, it is unknown whether EC cells respond directly to glucose in humans, and whether their capacity to release 5-HT is perturbed in obesity. We therefore investigated 5-HT release from human duodenal and colonic EC cells in response to glucose, sucrose, fructose and α-glucoside (αMG) in relation to body mass index (BMI). EC cells released 5-HT only in response to 100 and 300 mM glucose (duodenum) and 300 mM glucose (colon), independently of osmolarity. Duodenal, but not colonic, EC cells also released 5-HT in response to sucrose and αMG, but did not respond to fructose. 5-HT content was similar in all EC cells in males, and colonic EC cells in females, but 3 to 4-fold higher in duodenal EC cells from overweight females (*p* < 0.05 compared to lean, obese). Glucose-evoked 5-HT release was 3-fold higher in the duodenum of overweight females (*p* < 0.05, compared to obese), but absent here in overweight males. Our data demonstrate that primary human EC cells respond directly to dietary glucose cues, with regional differences in selectivity for other sugars. Augmented glucose-evoked 5-HT release from duodenal EC is a feature of overweight females, and may be an early determinant of obesity.

## 1. Introduction

Serotonin (5-hydroxytryptamine; 5-HT) is a monoamine produced from the hydroxylation of the dietary amino acid L-tryptophan via the rate-limiting actions of tryptophan hydroxylase (TPH). In vertebrates, two isoforms of TPH are encoded by the genes *TPH1* and *TPH2,* and give rise to independent pools of 5-HT. TPH1 is expressed in the periphery, largely in enterochromaffin (EC) cells of the gastrointestinal (GI) tract [1,2] where the majority (~90%) of total body 5-HT is produced [3]. TPH2 is expressed largely in neurons of the myenteric plexus, and centrally in the Raphe nuclei of the brainstem [2]. Gut-derived 5-HT enters the circulation and is primarily sequestered in platelets. However, free (extracellular) 5-HT can be transported into cells expressing the serotonin transporter (SERT). In the liver, this reuptake precedes breakdown of 5-HT to the metabolite *5*-hydroxyindoleacetic acid (5-HIAA), which is then excreted via the kidney. 

EC cells express a repertoire of receptors for sweet and bitter tastants, amino acids, fatty acids and bile acids, as well as transporters for nutrient absorption [4,5,6]. EC cells are also stimulated by short-chain fatty acids and secondary bile acids produced by gut microbiota [7] and by mechanical stimulation [8,9]. Glucose has been shown to directly trigger 5-HT release from isolated primary EC cells from guinea-pigs and mice [10,11], as well as from the intact rodent gut [12]. 5-HT released from the gut serves pleiotropic roles, including as a paracrine modulator of colonic motility [8,13,14], and activator of vagal afferent 5HT_3_ receptors to slow gastric emptying to optimise digestion and absorption of nutrients [15]. Other roles in the periphery include regulation of GI secretory responses, platelet aggregation and immune function [16,17,18], while centrally, 5-HT serves as a well-described neurotransmitter for mood, sleep, pain perception and appetite control [19]. Fasting also increases gut *Tph1* gene expression and plasma 5-HT levels in mice, which mobilises energy stores via stimulation of gluconeogenesis and lipolysis in hepatocytes and adipocytes, respectively, via 5-HTR_2B_ receptors [20]. 

Gut-derived 5-HT has recently emerged as a mediator of obesogenic processes. Plasma 5-HT and EC numbers in the proximal intestine are increased in rodent and human models of obesity, and in rodents this has been shown to occur ahead of weight gain [21,22,23,24], while genetic or pharmacological inhibition of TPH protects against diet-induced obesity and dysglycaemia in mouse models of obesity. This protection against obesity is in large part due to the attenuation of 5-HT-dependent inhibition of energy expenditure via adaptive thermogenesis [20,25].

While regional differences in nutrient sensing capability have been reported in EC cells in mice [11], there are no equivalent data in humans. Furthermore, it is unknown whether glucose-evoked 5-HT secretion from human EC cells differs between health and obesity, or between male and female subjects. In this study, we isolated EC cells from the duodenum and colon of non-obese and obese subjects to investigate sugar-dependent activation. We determined ex vivo EC cell responses to euglycemic (5 mM glucose), hyperglycemic (30 mM) and meal-related luminal glucose concentrations (100 mM, 300 mM), and responses to sucrose, fructose, α-methyl-D-glucopyranoside (αMG) and mannitol. We show that EC-5-HT secretion is dose-dependent at meal-related glucose concentrations, and that there were regional differences in EC cell glucose sensing and sugar selectivity. Finally, we show that 5-HT outputs from duodenum are augmented in overweight females, which may represent a sex-specific driver of obesity. 

## 2. Materials and Methods 

### 2.1. Subjects

For duodenal tissues, subjects aged ≥ 18 years were recruited from patients undergoing an endoscopic investigation of the upper gastrointestinal tract at the Royal Adelaide Hospital (RAH). Subjects fasted for endoscopy overnight, and had an intravenous cannula inserted into a forearm vein for administration of intravenous sedation (midazolam and fentanyl) prior to the procedure. Upper GI endoscopy (GIF-H180, Olympus, Tokyo, Japan) was performed to the second part of the duodenum, from which mucosal biopsies were collected using standard biopsy forceps. For colonic tissue, morphologically normal colonic tissue specimens were collected from patients undergoing bowel resections for cancer or stoma reversal at the Flinders Medical Centre and Flinders Private Hospital. Subjects who were pregnant, or who were receiving drugs known to alter gastrointestinal function were excluded from the study. Subjects were also excluded if mucosal abnormalities were detected at endoscopy or colon dissection. Study protocols were approved by the Human Research Ethics Committees of the RAH (131119), Flinders Medical Centre and Flinders Private Hospital (EC00188) and conducted in accordance with the Declaration of Helsinki, as revised in 2000. Written informed consent was obtained from all participants.

### 2.2. Cell Isolation

Duodenal and colonic tissue samples were collected into modified N-2-hydroxyethylpiperazine-N′-2-ethanesulfonic acid (HEPES) buffer (containing, in mM: 140 NaCl, 5 KCl, 2 CaCl_2_, 1 MgCl_2_, 10 HEPES, 5 Glucose, pH 7.4). EC cells were isolated from duodenal and colonic tissue as described [24,26]. For colon samples, mucosa was scraped from ~2cm^2^ of resected colon. Minced colonic mucosa and duodenal biopsies were digested for 45 min (duodenum) or 1 h (colon), prior to filtration and separation using a 10-step Percoll density gradient (density (d) = 1.024 to 1.123 g/mL) (GE Healthcare). Cells were harvested from the consecutive Percoll layers 1.047, 1.059 and 1.07 g/mL (cell density 1.047 g/mL < *d* < 1.082 g/mL), washed in growth medium, and incubated in fresh growth medium at 37 °C, 5% CO_2_ for between 1 and 2 h, until commencement of the secretion assay. Preparations of >90% EC cells (5-HT positive) are typically obtained using this method [10,11,24,26]. 

### 2.3. Secretion Assay, 5-HT Content and Sensitivity Measurements

Following isolation, cell counts, and viability were assessed using a hemocytometer and Trypan blue dye exclusion. Cells were centrifuged at 700× *g* for 5 min, the supernatant discarded, and cells washed and resuspended in Krebs containing 1uM fluoxetine (SERT inhibitor) and 5-HT stabilisation buffer (Labor Diagnostika Nord (LDN), Nordhorn, Germany) and pH adjusted prior to the assay. 120 µL of cell suspension (2.5 × 10^4^ viable cells) was placed in wells of a 96-well plate, and incubated at 37 °C with 5% CO_2_ for 30 min, to allow the cells to adhere and equilibrate. For secretion experiments, 60 µL was collected from each well and 3 of these samples stored to determine the average pre-treatment baseline 5-HT concentration. Care was taken at all stages to minimise mechanical stimulation of the EC cells. For response testing, 60 µL of treatment (in Krebs + 1µM fluoxetine (Sigma Aldrich, St. Louis, Missouri, USA) + 5-HT stabiliser (LDN, Nordhorn, Germany), pH 7.4) was added to each well to achieve the desired assay concentration for each sugar, and the cells were incubated for 20 min at 37 °C in 5% CO_2_. D-glucose, sucrose, methyl α-D-glucopyranoside (αMG), D-fructose, and D-mannitol were purchased from Sigma Aldrich, St. Louis, Missouri, USA. 80µL was then collected and stored at −20°C. From the 5-HT ELISA data, net 5-HT secretion was calculated by subtracting the average baseline reading from each final reading. 

For total 5-HT content measurements, cells were lysed after equilibration by replacing 110 µL Krebs with 110 µL of deionised H_2_O (containing a 1x 5-HT stabiliser (LDN, Germany), pH 7.4). A sample of 80 µL was collected after 20 min and stored at −20°C. 5-HT in cell secretions and EC cell lysates was determined by enzyme-linked immunosorbent assay (BAE-5900 Labor Diagnostika Nord, Nordhorn, Germany) and expressed in nM (2.5 × 10^4^ cells per 120µL). 

As a measure of EC sensitivity to glucose, the glucose-evoked 5-HT output:content ratio was calculated as the ratio of net secretion of 5-HT in 20 min exposure to 300 mM glucose to cellular 5-HT content in a parallel sample of cells at the zero timepoint. 

### 2.4. Statistics

**Figure 1:**
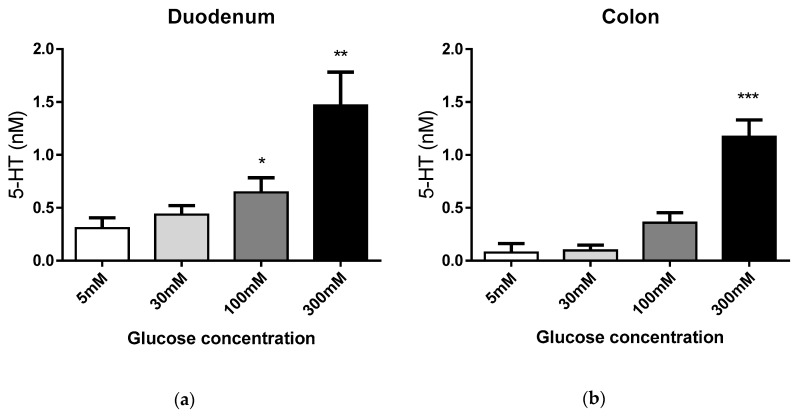
Human enterochromaffin (EC) cells respond to meal-related glucose concentration. Glucose-evoked 5-HT release from EC cells from human duodenum (**a**) and colon (**b**) is dose dependent in response to meal-related (luminal) glucose concentrations. Paired data shown for duodenum (*N* = 15) and colon (*N* = 17). * *p* < 0.05; ** *p* < 0.01, *** *p* < 0.001.

**Figure 2:**
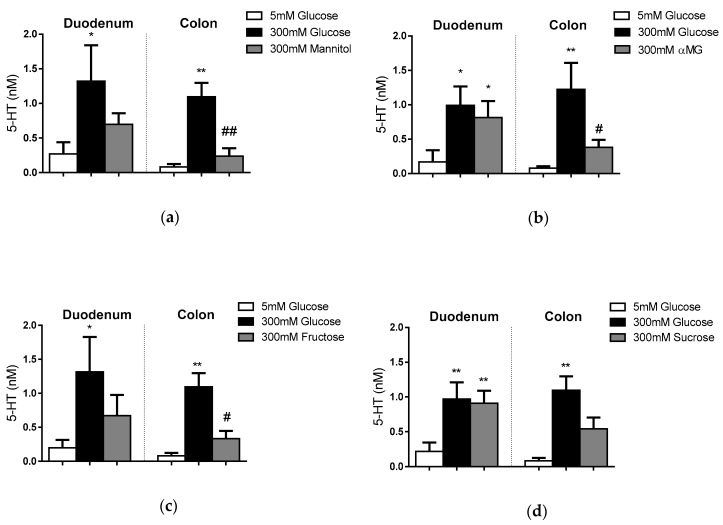
EC cell responses to glucose and other sugars. Release of 5-HT from duodenal and colonic EC cells in response to 300 mM glucose and equimolar (**a**) mannitol (*N* = 7 duodenum, 6 colon), (**b**) αMG (*N* = 5 duodenum, 9 colon), (**c**) fructose (*N* = 6 duodenum, 6 colon), and (**d**) sucrose (*N* = 7 duodenum, 6 colon). * represents significant differences compared to 5mM glucose, # represents significant differences compared to 300mM glucose; *p* < 0.05 (*, #), *p* < 0.01 (**, ##).

**Figure 3:**
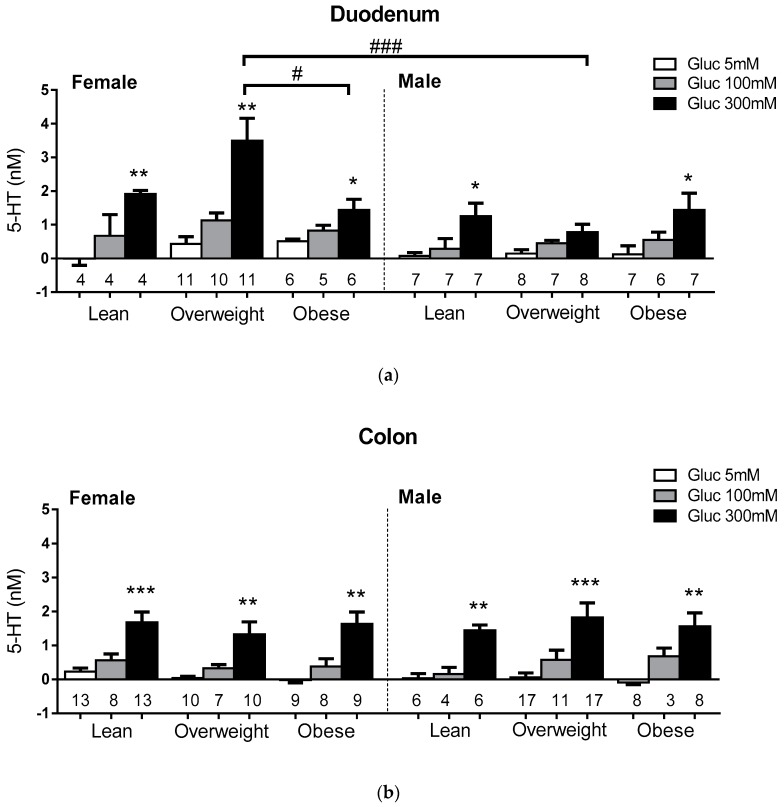
Glucose-evoked 5-HT secretion from human EC cells. Glucose-evoked responses of EC cells from (**a**) duodenum and (**b**) colon. The number of patients in each group (*n*) is shown under each bar. * represents significant differences compared to 5mM glucose, and # represents significant differences between BMI groups and between sexes. *p* < 0.05 (*, #), *p* < 0.01 (**), *p* < 0.001 (***, ###).

**Figure 4:**
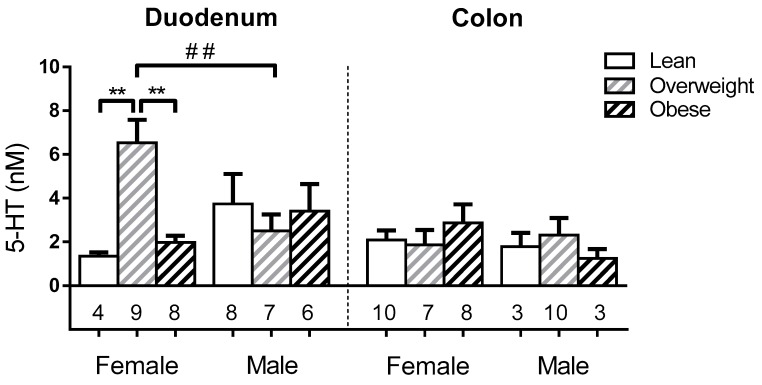
5-HT content is increased only in duodenal EC cells from overweight females. Comparison of the 5-HT content in EC cells by sex, BMI category, and gut region. The number of patients in each group (N) is shown under each bar. * and # represent significant differences in the duodenum, in a two-way ANOVA considering sex and BMI category with Holm-Sidak’s multiple comparison test. * represents significant differences between BMI categories; # represents significant differences between sexes. *p* < 0.01 (**, ##).

**Figure 5:**
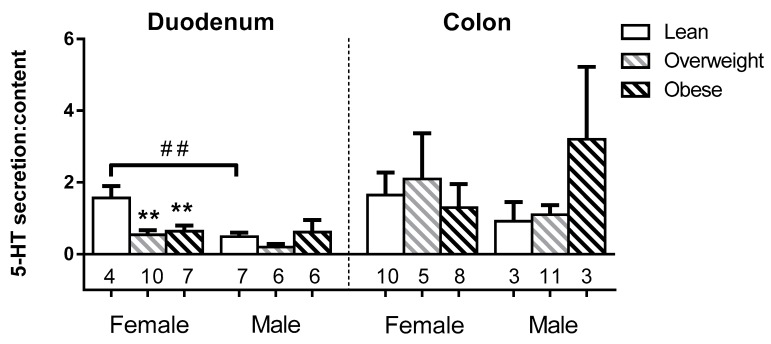
Duodenal EC cells from lean females are more sensitive to glucose-evoked 5-HT release than those from non-lean females, or lean males. The number of patients in each group (*N*) are shown under each bar. * represents significant BMI-related differences compared to the corresponding lean group; # represents significant differences between sexes. *p* < 0.01 (**, ##).

Statistical analysis was performed using Prism software (version 6; GraphPad, La Jolla, CA, USA). Sugar-dependent 5-HT outputs (Figure 1, Figure 2 and Figure 3) for each sex/BMI/gut region group were analysed using a paired one-way analysis of variance (ANOVA) with Tukey’s post-hoc test. To compare 5-HT content (Figure 4) and 300 mM glucose-evoked 5-HT release (Figure 3) considering BMI and sex as factors, a two-way ANOVA was used, with a Holm-Sidak’s post-hoc test. An unpaired 1-way ANOVA with Tukey’s post hoc test was used to compare glucose sensitivity for each sex and within each gut region (Figure 5). Data represents mean +/− SEM; Probability (*p*) values < 0.05 were considered significant. 

## 3. Results

### 3.1. EC Cell Responses to Extracellular Glucose

EC cells from non-obese subjects (BMI < 30 kg/m^2^) were used to investigate 5-HT glucose-evoked responses (Demographics, Table 1). EC cells were exposed to euglycemic 5 mM (control), hyperglycaemic 30 mM, and meal-related (luminal) 100 mM or 300 mM glucose concentrations for 20 min, and glucose-evoked 5-HT release determined. Duodenal EC cells released 5-HT in a dose-dependent manner at meal-related 100 mM (*p* < 0.05) and 300 mM glucose concentrations (*p* < 0.01), while 5-HT was released from colonic EC cells at 300 mM glucose (*p* < 0.001, Figure 1). No significant response was observed in 30 mM glucose (Figure 1). 

### 3.2. EC Cell Responses to Other Extracellular Sugars

The regional and nutrient-sensing repertoire of EC cells were determined in paired comparisons of 5-HT outputs in response to 300 mM glucose and equimolar mannitol (non-absorbable hexose, osmotic control), α-methyl-D-glucopyranoside (αMG; an absorbable, but non-metabolised, sugar derived from glucose), fructose and sucrose (Figure 2A–D). The release of 5-HT from duodenal and colonic EC cells did not occur in response to 300 mM mannitol (Figure 2A). Duodenal EC cells increased 5-HT release in response to 300 mM glucose (*p* < 0.05), αMG (*p* < 0.05) and sucrose (*p* < 0.01). In contrast, colonic EC cells released 5-HT only in response to glucose (*p* <0.01).

### 3.3. Sex and BMI Related Effects on EC 5-HT Content 

We assessed whether EC cell 5-HT content was related to sex or BMI category in lean (BMI < 25 kg/m^2^), overweight (25 kg/m^2^ ≤ BMI < 30 kg/m^2^) or obese (BMI ≥ 30 kg/m^2^) subjects (Demographics, Table S1). Duodenal EC cells from overweight females had > 3-fold higher 5-HT content (6.5 ± 1.0 nM) than EC cells from either lean or obese females (1.3 ± 0.2 nM; 2.0 ± 0.3 nM respectively; both *p* < 0.001), and ~2.6-fold higher levels than overweight males (2.5 ± 0.8 nM; *p* < 0.05). In contrast, EC cell 5-HT content was unrelated to BMI in males, and was similar in colonic EC cells for both sexes (Figure 3). 

### 3.4. Sex and BMI Related Effects on Glucose-Evoked 5-HT Release.

Glucose-evoked 5-HT release from EC cells in lean, overweight and obese subjects at euglycemia (5 mM glucose), and at meal-related glucose concentrations (100 mM, 300 mM), were compared in duodenum and colon (Demographics, Table S2). 5-HT release from duodenal and colonic EC cells was similar across lean, overweight and obese subjects, and sexes, at 5 mM glucose, and was augmented at 300 mM glucose in duodenum and colon in all groups relative to euglycemic lean (*p* < 0.05), except in the duodenum of overweight males (Figure 4). At 300 mM glucose, EC cell 5-HT outputs were 3-fold higher in the duodenum of overweight (3.5 ± 0.7 nM) compared to obese females (1.4 ± 0.3 nM; *p* < 0.05), and 4-fold higher than overweight males (0.8 ± 0.2 nM). 

### 3.5. Sex and BMI Related Effects on EC Cell Glucose Sensitivity

To investigate whether BMI-associated differences in glucose-evoked 5-HT output were related to altered glucose sensitivity, the ratio of 300mM glucose-evoked 5-HT released (in 20 min) to cellular 5-HT content (at time = 0) was calculated (Figure 5; Demographics, Table S3). A decrease in 5-HT output:content ratio was evident in EC cells from the duodenum of female subjects for both overweight (0.5 ± 0.1; *p* < 0.01) and obese (0.6 ± 0.2; *p* < 0.05) groups compared to the lean group (1.6 ± 0.3). This suggests that decreased glucose sensitivity accompanies the increased 5-HT synthesis (content) in the overweight group (Figure 3). No difference in output:content ratio was observed between the female overweight and obese groups. EC cells from lean female duodenum showed more release per content than those from lean male duodenum (0.5 ± 0.1 nM). No BMI-associated differences in output:content ratio were observed in colonic EC cells or duodenal EC cells from male subjects (Figure 5).

## 4. Discussion

This study is the first to describe a dose and glucose-dependent release of 5-HT from primary human EC cells in response to meal-related luminal glucose concentrations. We also showed that this response does not occur at a high post-prandial blood glucose concentration. We found that 5-HT release from duodenal EC cells was augmented, and more broadly tuned to sugars, than from colonic EC cells. All outputs were sugar-specific, and not triggered by iso-osmotic mannitol. Finally, we showed that duodenal EC cells from overweight female subjects had higher 5-HT content compared to lean and obese female subjects, and greater glucose-evoked 5-HT secretion than those from obese females. Furthermore, 5-HT content and glucose-evoked secretion were greater in EC cells from overweight females than from overweight males. Comparing the findings of this study with previous studies reveals similarities and differences in EC cell glucose sensing biology across species, and according to gut location. The finding that human EC cells release 5-HT in response only to luminal (and not serum) glucose concentrations is consistent with past investigations from our group, in guinea pig [10] and mouse [6]. However, although cross-species similarities in EC sugar selectivity were apparent between mouse and human, only human duodenal EC cells respond to αMG, while only mouse EC cells respond to fructose [11]. This highlights a broad range of glucose-sensing mechanisms in EC cells, which are specific to gut location and species. 

We show that duodenal and colonic EC cells respond to high glucose in humans, but with a broader repertoire of sugar sensing in the former. In vivo, these gut regions experience different luminal products and carbohydrate concentrations. The duodenum, like other small intestine regions, is tuned to dietary carbohydrate absorption, in contrast to the colon. The colon is unlikely to experience high glucose concentrations except in the setting of incomplete small intestine absorption, such as in rapid intestinal transit due to disease, or following resective surgery. Moreover, regional differences in nutrient receptor expression that may subserve these distinct sensing repertoires have been reported in mouse and human small and large intestine [5], and in isolated primary EC cells isolated from mouse duodenum and colon [11]. 

We observed the sucrose-dependent release of 5-HT in human duodenum. Sucrose is a putative ligand for the sweet taste receptor (comprised of T1R2 and T1R3 subunits), which is localised only to the small intestine in the gut of mice and humans [5]. The lack of response to fructose, however, argues against a major involvement of sweet taste receptors in the sugar sensing repertoire of human EC cells. This is in contrast to our earlier findings of duodenum-specific and fructose-dependent 5-HT release, and enrichment of T1R3 transcripts, in EC cells from mouse duodenum [6,11] where sweet taste receptors are likely to be involved in sugar sensing. An alternative possibility in humans is that the sucrose response is mediated by monosaccharide glucose liberated by the actions of disaccharidases present in the brush border membrane of the small intestine. 

The release of 5-HT from human duodenal EC cells in response to the glucose analogue αMG indicates that glucose metabolism is not required to trigger 5-HT release from human duodenal EC cells. Glucose and αMG are substrates of the sodium-glucose co-transporter family of glucose transporters (such as SGLT1), and sensors (SGLT3), that are abundantly expressed in the proximal intestine [27,28]. The selectivity of human duodenal EC cells for glucose and αMG, but not fructose, is similar to that of BON cells [29], a serotonergic human cell line derived from a pancreatic neuroendocrine tumour, in which SGLT1 is an identified glucose sensor [29]. Indeed, SGLT1 transcripts are present in EC cells from mouse and human [4,6], while SGLT1-dependent glucose transport in proximal (but not distal) L-cells triggers a glucose-dependent release of the incretin, glucagon-like peptide 1 (GLP-1) [30]. SGLT3 has also been proposed as a candidate glucose sensor in EC cells. This is based on evidence from rodent studies that 5-HT is released into the mesenteric lymph in response to glucose, but not galactose (an SGLT-1 selective substrate), and that EC cells are activated directly by the SGLT3 agonist, deoxynojirimycin [31,32]. However, the exact glucose sensor(s) in human EC cells remain to be identified.

While SGLTs have the potential to trigger glucose-evoked 5-HT release in proximal EC cells in humans, the lack of response of colonic EC cells to αMG (and previously, in mice) suggests against the involvement of SGLTs in the colon. While nutrient sensing in human EC cells has been investigated in EC cells of the proximal intestine [33], and pancreas-derived BON cells (which have proximal intestine-like properties with regards to sugar selectivity), our study is the first to report glucose-evoked 5-HT release from human intestinal EC cells, and also represents the first investigation of primary colonic EC cells with regards to nutrient sensing. Our findings support the existence of a distinct glucose-sensing pathway in colonic EC cells (that may co-exist in proximal cells) that, based on the lack of response to αMG, appears to require glucose metabolism.

Mounting evidence indicates that peripheral 5-HT production and bioavailability is increased in obesity. Expression of TPH1 [23,24] and PAX4 (a critical transcription factor in EC cell specification) [23] is augmented in the duodenum and gastric antrum of obese subjects, while levels of plasma 5-HT and serum levels of the 5-HT metabolite, 5-HIAA, are associated with measures of human obesity [24,34]. Rodent models of obesity also show increased EC cell density (per unit area) and 5-HT content, which precedes obesity, as well as augmented plasma 5-HT [21,22,23]. Indeed, peripheral pharmacological blockade or genetic disruption of TPH is protective against obesity and the development of diabetes in high fat diet-fed mice [20,25], highlighting a role for peripheral 5-HT in obesogenic processes. We recently reported that obese subjects have an augmented capacity to release gut derived serotonin in response to duodenal glucose infusion, relative to non-obese subjects (males and females) [24]. No differences in glucose sensitivity (proportion of EC cells activated by glucose) or cellular 5-HT content in isolated primary duodenal EC cells were observed, indicating that increased EC cell density was the main cause of the increased gut-5-HT capacity. Together with findings above, those findings are consistent with the view that increased EC cell density and tissue releasable 5-HT, rather than increases in cellular 5-HT content, augment circulating 5-HT in obesity.

Here we have investigated whether the capacity of EC cells to secrete 5-HT in response to glucose varies in relation to sex and BMI. We revealed that while colonic EC cells had similar content and glucose-evoked 5-HT release, duodenal EC cells in overweight females had higher 5-HT content than lean or obese, and augmented glucose-evoked 5-HT release compared to obese. In addition, while duodenal EC cells from lean and obese male subjects responded to glucose, glucose-evoked 5-HT release was not apparent in overweight male subjects.

An explanation for the increased capacity of the duodenal 5-HT system in overweight females may be that metabolic influences that demand and increase gut 5-HT in overweight individuals are mitigated through increases in EC cell density in established obesity. To this end, it is known that 5-HT synthesis in pancreatic islets is increased, and augments insulin secretion, for the metabolic demands of pregnancy [35] or in response to a high-fat diet [36]. The decreased glucose-evoked sensitivity of glucose-evoked 5-HT release in the duodenum of overweight and obese females may be a similar phenomenon to that occurring in pancreatic beta cells of individuals with type 2 diabetes. While the physiological ramifications of the loss of glucose sensitivity in these cohorts is unclear, it could potentially affect local 5-HT effects on chloride secretion and gut motility in response to nutrient ingestion. One possibility may be that metabolic influences that demand and increase gut 5-HT in overweight individuals are mitigated through increases in EC cell density in established obesity.

Increased intestinal 5-HT outputs in females may have implications not only for the endocrine effects of 5-HT on metabolism [37], but also for gut paracrine 5-HT signalling. Relatively small amounts of 5-HT are released from guinea-pig primary colonic EC cell vesicles per exocytic event [38,39,40,41] and acute exposure to high glucose (100 nM) increases the amount of serotonin released per fusion event, without affecting the frequency of exocytic events [10]. The amount released per fusion event is dictated by several factors, including vesicle loading. Increased 5-HT vesicle loading will augment activation of adjacent 5-HT receptors on afferent nerve terminals [26], which may, correspondingly, increase gut motility and slow gastric emptying. Interestingly, many patients with gastroparesis, a condition associated with markedly slowed or incomplete gastric emptying, are overweight females [42]. Our findings of increased 5-HT synthesis in EC cells from overweight females offer a potential explanation for the sex bias in gastroparesis, via a mechanism involving downstream 5-HT signalling effects on gut motility and/or vagal activation.

We observed lower glucose-evoked 5-HT release in obese compared to overweight females. While rodent and human studies provide evidence of increased gut derived 5-HT in obesity (as described above), a loss of luminal glucose-evoked 5-HT release has been identified as an early event in diet-induced obesity in rats [43]. Specific reduction in EC cell glucose-evoked 5-HT output (compared to overweight, but not too lean) in obese female (human) subjects has not been previously described. Sex-specific differences in intestinal 5-HT physiology exist in rodents, with higher EC cell density and increased jejunal 5-HT availability reported in female mice, and a male-specific impairment of SERT-mediated 5-HT reuptake in the small intestine in diet-induced obesity [21,44]. Evidence suggests that this impairment of SERT is accompanied by an upregulation of the dopamine transporter (DAT) [44]. In our study, duodenal EC cells did not show glucose-dependent 5-HT release in overweight males. Whilst a SERT inhibitor was used in our assays to prevent serotonin reuptake, we cannot exclude the possibility of reuptake via DAT. 

Due to the opportunistic nature of specimen acquisition, we did not collect information on the hormonal status of female patients, which should be investigated using a more targeted approach in future studies. However, levels of plasma sex hormones estradiol, progesterone and testosterone, were unaffected by high-fat diet feeding in mice that exhibited sex-biased differences in serotonergic homeostasis [44]. The sex-specific nature of these mechanisms in humans requires further study. 

## 5. Conclusions

In conclusion, we showed that primary human EC cells secrete 5-HT in response to meal-related luminal concentrations of glucose, with BMI and region dependent variability in 5-HT content and output in females. Broader selectivity for sugars in the proximal intestine supports the presence of multiple sensing pathways, whilst glucose specificity in the distal intestine raises the possibility of previously uncharacterised glucose sensing mechanism(s). Future research should focus on investigating the cellular mechanisms of glucose sensing and stimulus-secretion pathways in human EC cells. Understanding the drivers behind EC cell 5-HT synthesis, and release in overweight females, and its physiological relevance, will provide new insight into the expanding roles of gut 5-HT in health and disease. 

## Figures and Tables

**Table 1:** Demographics for glucose dose response in non-obese individuals for data shown in Figure 1.

	Duodenum	Colon
***n***	15	17
**BMI (kg/m^2^)**	24.9 ± 0.9	25.0 ± 0.9
**Age (years)**	50.2 ± 5.4	69.6 ± 4.4
**M:F**	10:5	6:11

## Supplementary Materials

The following are available online at http://www.mdpi.com/2072-6643/11/2/234/s1, Table S1: Demographics for enterochromaffin cell 5-HT content in Figure 3, Table S2: Demographics for 300 mM Glucose response in Figure 4, Table S3: Demographics for glucose sensitivity in Figure 5.
