# Supplementary material for: Sugar Responses of Human Enterochromaffin Cells Depend on Gut Region, Sex, and Body Mass
Source: Nutrients. 2019 Jan 22;11(2):234. doi: 10.3390/nu11020234 (PMC6412251; doi:10.3390/nu11020234)
Supplement: Supplementary file 1 [file nutrients-11-00234-s001.zip › Table S1.docx]

**Table S1.** Demographics for enterochromaffin cell 5-HT content in Figure 3.

|  |  | **Female** | | | | **Male** | | |
| --- | --- | --- | --- | --- | --- | --- | --- | --- |
|  |  | **Lean** | **Overweight** | | **Obese** | **Lean** | **Overweight** | **Obese** |
| **DUO** | *n* | 4 | | 9 | 8 | 8 | 7 | 6 |
|  | BMI (kg/m^2^) | 20.6 ± 0.5 | | 27.7 ± 0.5 | 35.3 ± 1.3 | 22.0 ± 0.8 | 26.7 ± 0.4 | 31.1 ± 0.7 |
|  | Age (years) | 57.0 ± 14.0 | | 56.4 ± 7.0 | 59.9 ± 3.4 | 39.7 ± 6.9 | 46.5 ± 4.5 | 64.3 ±1.9 |
| **COLON** | *n* | 10 | | 7 | 8 | 3 | 10 | 3 |
|  | BMI (kg/m^2^) | 21.9 ± 0.5 | | 28.0 ± 0.5 | 35.0 ± 1.4 | 23.1 ± 1.6 | 27.4 ± 0.5 | 37.1 ± 5.0 |
|  | Age (years) | 70.6 ± 5.0 | | 61.4 ± 9.1 | 53.3 ± 7.3 | 75.7 ± 6.6 | 65.0 ± 3.7 | 59.0 ± 7.8 |
