# Supplementary material for: Sugar Responses of Human Enterochromaffin Cells Depend on Gut Region, Sex, and Body Mass
Source: Nutrients. 2019 Jan 22;11(2):234. doi: 10.3390/nu11020234 (PMC6412251; doi:10.3390/nu11020234)
Supplement: Supplementary file 1 [file nutrients-11-00234-s001.zip › Table S2.docx]

**Table S2.** Demographics for 300mM Glucose response in Figure 4.

|  |  | **Female** | | | **Male** | | |
| --- | --- | --- | --- | --- | --- | --- | --- |
|  |  | Lean | Overweight | Obese | Lean | Overweight | Obese |
| **DUO** | *n* | 4 | 11 | 6 | 7 | 8 | 7 |
|  | BMI (kg/m^2^) | 20.6 ± 0.5 | 27.5 ± 0.4 | 36.5 ± 1.4 | 22.2 ± 0.9 | 27.1 ± 0.5 | 31.0 ± 0.6 |
|  | Age (years) | 57.0 ± 14 | 60.0 ± 6.2 | 61.7 ± 4.3 | 35.2 ± 6.0 | 48.0 ± 4.2 | 64.4 ±1.6 |
| **COLON** | *n* | 13 | 10 | 9 | 6 | 17 | 8 |
|  | BMI (kg/m^2^) | 22.0 ± 0.5 | 27.8 ± 0.4 | 34.7 ± 1.0 | 23.2 ± 0.7 | 27.3 ± 0.3 | 34.7 ± 1.9 |
|  | Age (years) | 73.0 ± 4.2 | 65.6 ± 6.7 | 48.7 ± 6.0 | 70.0 ± 5.5 | 68.1 ± 2.9 | 60.9 ± 4.8 |
