# Supplementary material for: Sugar Responses of Human Enterochromaffin Cells Depend on Gut Region, Sex, and Body Mass
Source: Nutrients. 2019 Jan 22;11(2):234. doi: 10.3390/nu11020234 (PMC6412251; doi:10.3390/nu11020234)
Supplement: Supplementary file 1 [file nutrients-11-00234-s001.zip › Table S3.docx]

**Table S3.** Demographics for glucose sensitivity in Figure 5.

|  |  | **Female** | | | **Male** | | |
| --- | --- | --- | --- | --- | --- | --- | --- |
|  |  | **Lean** | **Overweight** | **Obese** | **Lean** | **Overweight** | **Obese** |
| **DUO** | *n* | 4 | 10 | 7 | 7 | 6 | 6 |
|  | BMI (kg/m^2^) | 20.6 ± 0.5 | 27.7 ± 0.4 | 35.7 ± 1.4 | 22.4 ± 0.8 | 26.7 ± 0.4 | 31.1 ± 0.7 |
|  | Age (years) | 57.0 ±14 | 58.8 ± 6.7 | 60.1 ± 4.0 | 41.9 ± 7.5 | 47.0 ± 5.3 | 64.3 ± 1.9 |
| **COLON** | *n* | 10 | 6 | 8 | 3 | 11 | 3 |
|  | BMI (kg/m^2^) | 21.9 ± 0.5 | 27.9 ± 0.6 | 35.0 ± 1.4 | 21.2 ± 1.6 | 27.2 ± 0.5 | 37.1 ± 5.0 |
|  | Age (years) | 70.6 ± 5.0 | 61.7 ± 10.7 | 53.3 ± 7.3 | 79.7 ± 2.9 | 64.8 ± 3.4 | 59.0 ± 7.8 |
